# Infection rates associated with epidural indwelling catheters for seven days or longer: systematic review and meta-analysis

**DOI:** 10.1186/1472-684X-6-3

**Published:** 2007-04-04

**Authors:** Wilhelm Ruppen, Sheena Derry, Henry J McQuay, R Andrew Moore

**Affiliations:** 1 Department of Anaesthetics, University Hospital of Basel, Hebelstrasse 32, CH-4031, Basel, Switzerland; 2 Pain Research and Nuffield Department of Anaesthetics, University of Oxford, Oxford Radcliffe NHS Trust, The Churchill, Headington, Oxford, OX3 7LJ, UK

## Abstract

**Background:**

To determine infection rate with use of epidural catheters in place for seven days or more.

**Methods:**

Systematic review and pooled analysis of observational studies.

**Results:**

Twelve studies with 4,628 patients (median 197 patients) provided information, of which nine (4,334 patients) were published after 1990. Eight studies (3,893 patients) were retrospective, and four studies (735 patients) prospective. Electronic searches identified three studies and searching reference lists nine.

There were 257 catheter-related infections in total, of which 211 were superficial and 57 deep, giving rates of 6.1%, 4.6% and 1.2% respectively. Ten of the 12 studies had deep infection rates of 2% or less. The incidence of deep infection was 1 per 2391 days of treatment, or 0.4 per 1000 catheter treatment days. In nine studies (1503 patients), predominantly in cancer, and with average catheter duration of 74 days, the deep infection rate was 2.8%. The proportion of patients with infection of any type was higher in cancer patients with longer catheter duration. Limited numbers of events meant that no reliable estimate of the impact of prospective and retrospective design could be made. There appeared to be a relationship between catheter duration and infection rate from this and other recent estimates. Four of 57 (7%) patients with deep infection died.

**Conclusion:**

The best estimate is that one person in 35 with an epidural catheter in place for 74 days for relief of cancer pain can be expected to have a deep epidural infection, and that about 1 in 500 may die of infection-related causes. This is a most uncertain estimate given the limited nature of the evidence.

## Background

Managing very severe pain is important and not always easy. Not all patients receiving appropriate conventional analgesics for nociceptive pain experience adequate pain relief, and some suffer from intolerable adverse effects. Changing delivery route from oral to epidural for opioids is one strategy in this situation [1,2]. Epidural drug delivery may also be used to manage severe neuropathic or movement-related pain.

A potential risk of epidural catheters is infection, and infection in the epidural space can be a very serious complication [3]. The clinical decision to implant an epidural catheter is influenced by the predicted benefit and the risk of complications. At present we do not have robust estimates for the risk of infection in this situation.

Rates associated with short-term use of catheters in obstetrics have been calculated [4]: epidural haematoma 1 in 168,000 women, deep epidural infection 1 in 145,000, persistent neurological injury 1 in 240,000, and transient neurological injury 1 in 6,700. In obstetrics, though, catheters would often be in place for less than a day, while in cancer patients they may be in place for months. The aim of this meta-analysis was to determine the rates of infection for epidural catheters in place for seven days or longer.

## Methods

Papers reporting on adverse events associated with epidural catheters were identified using three different approaches. First we carried out electronic searches in PubMed (from 1966), MEDLINE (from 1966) and EMBASE (from 1980) to February 2005, with no language restrictions. The searches combined controlled vocabulary and free text terms for both the intervention (epidural catheter) and the outcome (adverse effect). Details of the terms used are in additional file 1. Secondly we hand searched five anaesthesia journals (Anesthesiology, Anesthesia and Analgesia, British Journal of Anaesthesia, Anaesthesia, Acta Anaestheseiologica Scandinavica) from mid 1999 to February 2005. Thirdly, reference lists of reviews and retrieved studies were checked for additional studies.

The titles and abstracts of all retrieved articles were read, and those clearly not relevant were eliminated. Full copies of all the remaining studies were obtained and read. Those reporting numerical data for serious adverse events were included in an initial list of studies. We then selected those reporting on at least 50 patients, with median catheter duration of at least seven days, and with numerical data for superficial infections (skin around catheter insertion), deep infections (in the epidural space), or total infections (deep and superficial), using the infection criteria used by the original authors.

Information about the type of study, patients, intervention, and numbers experiencing individual adverse outcomes was tabulated. QUOROM guidelines [5] were followed where applicable. It was the intention, where there was sufficient clinical homogeneity, to pool results and to calculate infection rates for deep and superficial infections separately. We planned to perform sensitivity analyses for larger versus smaller studies, studies published before or after 1990 to reflect possible changes in practice, and for perioperative patients and cancer patients separately. Information on infections would be presented in two ways: the percentage of patients with deep, superficial, or any catheter-related infection, and the incidence of infection per 1000 catheter treatment days.

## Results

**Figure 1:**
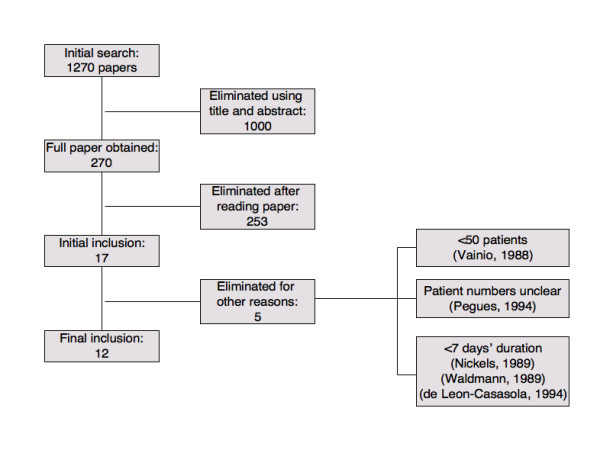
Flow chart of studies.

Initially 1,270 papers were identified, 270 of which referred to epidural harm. Seventeen appeared to relate to epidural catheters in place for at least seven days. We excluded five of these papers (Figure 1), three [6-8] because they were of short duration, one [9] had no denominator, and one [10] provided information on only 30 patients. Only three [2,11,12] of the 12 remaining studies were identified by electronic searches, and nine [1,13-20] were found from reference lists. One study [17] with 110 patients included 30 with subarachnoid injections, but no deep infections; because the numbers of subarachnoid injections were tiny compared with the total number of patients in the review, we included it.

Nine studies examined mainly cancer patients (1,481 cancer, 11 AIDS, 11 non-cancer), two [19,20] examined postoperative patients, though including some patients with cancer, and one [12] examined only patients with chronic non-cancer pain (Table 1).

**Table 1:** Detailed results from individual studies

					**Deep infection**			**Superficial infection**			**Any catheter-related infection**		
					
**Study**	**Patients**	**Cohort**	**Number of patients**	**Catheter duration (days)**	**Number with infection**	**Percent of patients affected**	**Per 1000 catheter days**	**Number with infection**	**Percent of patients affected**	**Per 1000 catheter days**	**Number with infection**	**Percent of patients affected**	**Per 1000 catheter days**
Aldrete 1998	Noncancer	Retrospective	504	2 to 80 days	4	0.8	no data	5	1.0	no data	9	1.8	no data
Cherry 1985	Cancer	Prospective	50	4200	0	0.0	0.0	2	4.0	0.5	2	4.0	0.5
Crawford 1983	Cancer (94) and benign (11) pain	Retrospective	105	6825	1	1.0	0.1	no data			no data		
Crul 1991	Cancer	Retrospective	110	8650	0	0.0	no data	5	4.5	no data	5	4.5	no data
de Jong 1994	Cancer	Retrospective	149	6969	3	2.0	0.4	no data			no data		
Du Pen 1990	Cancer (339) and AIDS (11)	Retrospective	350	32354	23	6.6	0.7	30	8.6	0.9	53	15.1	1.6
Erdeine 1991	Cancer	Prospective	225	10642	0	0.0	0.0	9	4.0	0.8	9	4.0	0.8
Holt 1995	Mixed	Prospective	1000	11901	11	1.1	0.9	53	3.6	3.0	59	5.9	5.0
Maier 1994	Perioperative	Retrospective	1621	12540	0	0.0	0.0	46	2.8	3.7	46	2.8	3.7
Plummer 1991	Cancer	Retrospective	284	27264	1	0.4	0.0	22	7.7	0.8	23	8.1	0.8
Smitt 1998	Cancer	Retrospective	91	4326	12	13.2	2.8	39	42.9	9.0	51	56.0	11.8
Zenz 1985	Cancer	Prospective	139	9716	2	1.4	0.2	no data			no data		

**Patients in studies reporting the outcome**					4628			4235			4235		

**Overall result**			4628	135387	57	1.23	0.42	211	4.58	1.78	257	6.07	2.35

The 12 papers involved 4,628 patients having an epidural catheter for at least seven days (full details of studies in additional file 2). The number of patients in each study ranged from 50 to 1621, median 197. Nine studies with 4,334 patients were published after 1990, and three with 294 patients before 1990 (Table 1); two studies published after 1990 [19,20] accounted for more than half of the total patients (2,621 patients). Eight studies (3,214 patients, at least 99,000 catheter days of follow up) were retrospective, two of which examined non-cancer patients [12,19]; four studies were prospective (1,414 patients, 36,000 catheter days [1,14,15,20]), one of which [20] examined non-cancer patients.

One study [12] did not indicate the mean catheter duration, and another [17] included 30 patients with subarachnoid injections. In the remaining 10 studies with 4,014 patients, the mean catheter duration for each study ranged from seven to 96 days, with an overall mean of 57 days. The total number of catheter days in each study ranged from 4,200 to 32,354. The overall number of patient days was more than 135,000; of these, 111,000 were obtained in cancer studies and of those, 36,000 were in three prospective studies and 78,000 in five retrospective studies.

### Infection rates

**Figure 2:**
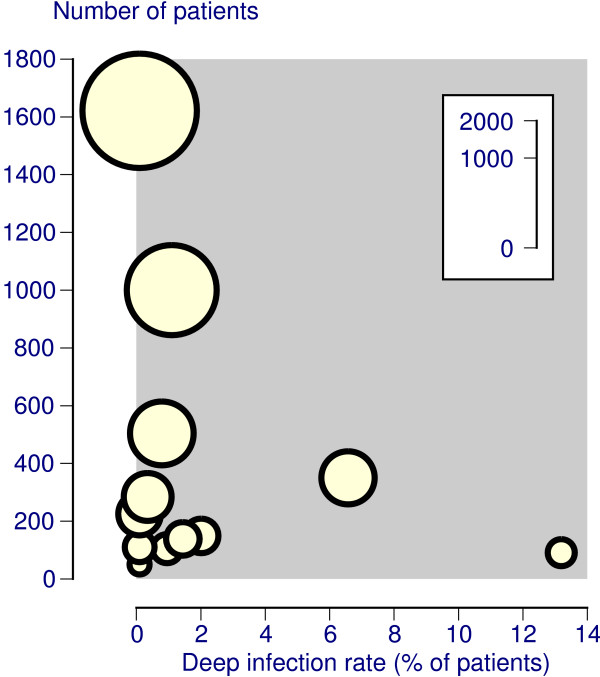
Deep infection rates in individual studies (size of symbol proportional to number of patients; inset scale).

Fifty-seven deep infections were reported in 12 studies with 4,628 patients (Table 1), giving a deep infection rate of 1.2% (95% CI 0.91 to 1.6) (Table 2). There was considerable variation between individual studies (Figure 2), though 10 of the 12 studies had deep infection rates of 2% or less. The incidence of deep infection was 1 per 2391 days of treatment, or 0.4 per 1000 catheter treatment days.

**Table 2:** Main results for deep, superficial and any infection

	**Site of infection**		
	
	**Deep**	**Superficial**	**Any catheter-related**
	
Number of studies	12	9	9
Number of patients with infections	57	194	257
Total number of patients	4628	4235	4235
Percentage of patients with infection	1.2	4.6	6.1
Risk of infection per 1000 catheter days	0.4	1.8	2.4
Mean days of catheter use before one infection occurs	2391	561	425

Note that the infection rate for deep plus superficial numbers and percentages does not necessarily equal that for any catheter-related infection because of different numerators and denominators

There were 211 superficial infections in nine studies with a total of 4,235 patients (Table 1), giving an overall infection rate of 4.6% (95% CI 4.0 to 5.2) (Table 2). Only one study had a superficial infection rate above 10% (Table 1). The incidence for superficial infection was 1 per 561 days of treatment or 1.8 per 1000 catheter treatment days.

We identified catheter-related infections of any description in 257 patients in the same nine studies (Table 1) giving an overall infection rate of 6.1% (95% CI 5.4 to 6.8) (Table 2). Overall infection rates were above 10% in two studies (Table 1). The incidence for any catheter-related infection was 1 per 425 days of treatment or 2.4 per 1000 catheter treatment days. Numbers and percentages for deep and superficial infections do not equal any catheter-related infection because of different numerators and denominators from different studies. We did not include three studies reporting only deep infections in the any catheter-related infection column (Table 1), since these were a small proportion of the total infections. In addition, one study [20] reported five patients with generalised symptoms of infection who did not have local infection. These 11 patients help explain the difference in numbers between deep plus local infections (57+211 = 268) and any catheter-related infections (257).

### Sensitivity analyses

Three studies [1,19,20] reported principally or wholly on non-cancer or perioperative patients. The average catheter duration in two of them was 9.3 days, and in the third the duration was between two and 80 days [12]. These three studies were also the largest studies, with more than 500 patients each (3,125 patients, 68% of the total; 25,000 catheter days, 19% of the total). The other nine studies were smaller, with between 50 and 350 patients each (1,503 patients, 22% of the total; 111,000 catheter days 81% of the total), and reported predominantly on cancer patients with average catheter duration of 74 days. Sensitivity analysis compared the three larger studies of shorter catheter duration in patients without cancer with the nine smaller studies of longer catheter duration in patients with cancer, and prospective and retrospective studies within the nine cancer studies (Table 3).

**Table 3:** Sensitivity analysis

		**Larger studies Postoperative, noncancer**		**Smaller studies Predominantly cancer**		**Prospective cancer studies**		**Retrospective cancer studies**	
		
**Infection**	**Outcome**	**patients**	**Result**	**patients**	**Result**	**patients**	**Result**	**patients**	**Result**
Deep infection	Percent of patients	15/3125	0.48	42/1503	2.8	2/414	0.48	39/979	4.0
	Incidence per 1000 days		0.45		0.41		0.08		0.50
Superficial infection	Percent of patients	87/3125	2.8	107/1110	9.6	11/275	4.0	61/725	8.4
	Incidence per 1000 days		3.4		1.3		0.74		0.95
Overall infection	Percent of patients	114/3125	3.6	143/1110	13	11/275	4.0	74/725	10
	Incidence per 1000 days		4.3		1.8		0.74		1.2

For deep, superficial, or overall infection, the smaller studies with long catheter duration in cancer had higher infection rates, some three or four times higher than the larger studies with a shorter catheter duration in mainly non-cancer patients. For instance, the deep infection rate in the larger, shorter, studies was 0.48%, while in the longer studies in cancer it was 2.8%; one cancer patient in 35 with a long duration epidural catheter would have a deep infection. The incidence per 1000 catheter days for superficial and deep infection in these smaller studies was a half to a third that in larger studies (Table 3). In the longer, cancer, studies the small number of events in the three prospective studies (one of which [15] had information only on deep infection) meant that no reliable estimate of effects of study design could be made (Table 3).

### Outcome with deep infection

Outcomes of infections were reported in six studies [2,11,12,15,16,18]. One of them [18] reported that one of three patients with a deep infection died. Another [2] reported that three of twelve with deep infections died, while the remaining nine had a good outcome. The other four studies reported the outcome of patients with superficial and/or deep infections as good. Overall, four of 57 patients (7%; 1 in 14 patients) with deep infections died, although we cannot be certain that the cause of death was solely due to the epidural infection. Good outcomes occurred in 41 of 57 patients (72%), and the outcome in 12 patients (21%) was not reported.

If the risk estimate of deep epidural infection in cancer patients with long duration catheters is 1 in 35 patients, and the risk of death from deep epidural infection is 1 in 14, then an overall risk of death from deep epidural infection in cancer patients with long duration catheters is of the order of 1 in 500.

## Discussion

A recent systematic review of efficacy of epidural, subarachnoid, and intracerebroventricular opioids for cancer pain [21] had information on 2,402 patients in 72 uncontrolled studies reporting on efficacy. It found eight spinal infections, without detailed description, in 154 patients (5.2%). By contrast, the present systematic review concentrated on reports of epidural infection, and found 57 cases of deep epidural infection in 4,628 patients (1.2%) in 12 uncontrolled studies. In comparing these two approaches, only one study (published as an abstract) included in the Ballantyne & Carwood review [21] was missed by our approach, and that reported only a single superficial infection in 129 patients. Several studies included in this review may have been eligible for that of Ballantyne & Carwood. Using adverse event information found solely in studies of efficacy appears to have restricted the amount of information available on serious adverse events.

Information was available for adverse events associated with the use of epidural catheters in 1.4 million women during childbirth [4] and the short duration of catheter use during childbirth minimised the likelihood of deep infection, occurring in 1 in 145,000 women (7 per million). In 14,105 patients undergoing cardiothoracic or vascular surgery with epidural catheters, no deep epidural infections were noted [22]. Results from situations where epidural catheters are used for only a few days are unlikely to be representative for those where catheter use is for months.

When catheters were in place for more than seven days in this chronic setting, deep infection occurred in 1 in 83 patients, 1,800 times more frequently than in obstetrics. This may be related to catheter duration, because sensitivity analysis (Table 3) demonstrated that studies of catheter use of shorter catheter duration (generally less than 10 days) had a deep infection rate of 0.48%, but when average catheter duration was 74 days the deep infection rate was 2.8%. While a strong link between catheter duration and deep infection rate may be inferred, differing case mix may also be relevant. The higher rate was in predominantly cancer patients, who are usually older and with clinical problems that might make infection more likely, though it was interesting (Table 3) that deep infection rates per 1,000 patient days in cancer patients were much the same as in mainly preoperative patients.

The best estimate we have, then, is that 1 in 35 cancer patients with long duration epidural catheters will have a deep epidural infection, and that about 1 in 500 may die of infection-related causes. The small number of cases and deaths, lack of information on cause of death, and issues like use of prophylactic antibiotics or predisposing clinical factors make this a most uncertain estimate.

The fact that the percentage of patients with deep epidural infection increased with catheter duration while the incidence per 1000 catheter days fell is not inconsistent. The reason is that longer duration catheters contribute more to the number of catheter days. Studies of short catheter duration had an average of about 10 days per patient or less, while those of longer duration had catheter duration per patient more than seven times longer. There would have to be seven times more patients with an infection for the incidence per 1000 patient days to be the same, but deep epidural infections actually increased only five-fold. In addition, the original studies are generally silent on issues like use of prophylactic antibiotics, which might contribute to some patients having long catheter placement without infection.

While epidural infection is undoubtedly a serious event, the outcomes are not always disastrous. Nussbaum et al [3] reported that 26 of 40 patients (65%) with an epidural abscess had a good, 9 (23%) a fairly good, and 5 (12%) a bad outcome. This is similar to outcomes after deep epidural infection in this review, with a good outcome in 72% and a bad outcome in 7%. A recent prospective study [23] concerning meningitis outcome after paravertebral injections in eight patients showed a favorable outcome in three, handicap in three, and death in one.

There are several weaknesses in this review, not least the relatively small number (57) of deep epidural infections, increasing the possible effects of chance [24]. It was not possible to identify concomitant risk factors that might suggest which patient is most at risk, how infections in these patients might be prevented, rates of progression from superficial to deep infection, or possible advantages of using tunneled catheters. Because cultural, legal, or personal considerations may affect reporting, serious adverse events may also be under-reported. Limited numbers of events also meant that no reliable estimate of the impact of prospective and retrospective design could be made, and information comes predominantly from retrospective studies.

A further limitation is that a variety of epidural systems (percutaneous, ports, tunnelled silastic and tunnelled polyamide) could have been used. It may be that they were associated with different infection rates, but with the small numbers of actual infections, it would have not been possible to come to any reliable conclusions with the data available.

Finally, searching for observational studies is more difficult than for randomised trials, despite considerable efforts using electronic databases. Electronic searching of a single database yielded only 60 to 80% of relevant observational studies [25]. Hand searching journals, and checking of reference lists of retrieved papers increases retrieval of relevant papers. We looked for studies in electronic databases, handsearched five main anaesthesia journals, and checked the reference lists of all included studies, and many excluded studies and reviews. Most (9/12) of the studies identified for this review were found in reference lists of previously retrieved articles and reviews, and 270 papers had to be read and their reference lists examined to ensure as complete a survey as possible. Only three relevant studies were found in electronic searches.

Particular care should be taken to avoid catheter implantation when there is either local (skin infection at puncture site) or systemic signs of infection. Ongoing steroid therapy may also predispose to epidural infection. When catheter placement is likely to be prolonged, special care needs to be given to catheter hygiene.

The results of this systematic review provide the best, if limited, available estimate for epidural infections in patients with long-term epidural catheters for analgesia. Larger numbers for both numerator and denominator would improve the strength of the estimate, and larger prospective consecutive series would be welcomed.

## Conclusion

The best estimate is that one person in 35 with an epidural catheter in place for 74 days for relief of cancer pain can be expected to have a deep epidural infection, and that about 1 in 500 may die of infection-related causes.

## Competing interests

The author(s) declare that they have no competing interests.

## Authors' contributions

RAM, HJM, and WR were involved with the original concept and planning the study. WR and SD did data extraction and analysis. WR and RAM prepared the initial manuscript, and all authors read and approved the final manuscript.

## Pre-publication history

The pre-publication history for this paper can be accessed here:



## Supplementary Material

Additional file 1 Search strategy Click here for file

Additional file 2 Details of included studies Click here for file

## References

[B1] Erdine S, Aldemir T (1991). Long-term results of peridural morphine in 225 patients. Pain.

[B2] Smitt PS, Tsafka A, Teng-van de Zande F, van der Holt R, Elswijk-de Vries I, Elfrink E, van den Bent MJ, Vecht CJ (1998). Outcome and complications of epidural analgesia in patients with chronic cancer pain. Cancer.

[B3] Nussbaum ES, Rigamonti D, Standiford H, Numaguchi Y, Wolf AL, Robinson WL (1992). Spinal epidural abscess: a report of 40 cases and review. Surg Neurol.

[B4] Ruppen W, Derry S, McQuay H, Moore RA (2006). Incidence of epidural hematoma, infection and neurological injury in obstetric patients with epidural analgesia/anesthesia: meta-analysis. Anesthesiology.

[B5] Moher D, Cook DJ, Eastwood S, Olkin I, Rennie D, Stroup DF (1999). Improving the quality of reports of meta-analyses of randomised controlled trials: the QUOROM statement. Quality of Reporting of Meta-analyses. Lancet.

[B6] Nickels JH, Poulos JG, Chaouki K (1989). Risks of infection from short-term epidural catheter use. Reg Anesth.

[B7] Waldman SD (1989). Complications of cervical epidural nerve blocks with steroids: a prospective study of 790 consecutive blocks. Reg Anesth.

[B8] de Leon-Casasola OA, Parker B, Lema MJ, Harrison P, Massey J (1994). Postoperative epidural bupivacaine-morphine therapy. Experience with 4,227 surgical cancer patients. Anesthesiology.

[B9] Pegues DA, Carr DB, Hopkins CC (1994). Infectious complications associated with temporary epidural catheters. Clin Infect Dis.

[B10] Vainio A, Tigerstedt I (1988). Opioid treatment for radiating cancer pain: oral administration vs. epidural techniques. Acta Anaesthesiol Scand.

[B11] Plummer JL, Cherry DA, Cousins MJ, Gourlay GK, Onley MM, Evans KH (1991). Long-term spinal administration of morphine in cancer and non-cancer pain: a retrospective study. Pain.

[B12] Aldrete JA, Williams SK (1998). Infections from extended epidural catheterization in ambulatory patients. Reg Anesth Pain Med.

[B13] Crawford ME, Andersen HB, Augustenborg G, Bay J, Beck O, Benveniste D, Larsen LB, Carl P, Djernes M, Eriksen J, Grell AM, Henriksen H, Johansen SH, Jorgensen HO, Moller IW, Pedersen JE, Ravlo O (1983). Pain treatment on outpatient basis utilizing extradural opiates. A Danish multicentre study comprising 105 patients. Pain.

[B14] Cherry DA, Gourlay GK, Cousins MJ, Gannon BJ (1985). A technique for the insertion of an implantable portal system for the long-term epidural administration of opioids in the treatment of cancer pain. Anaesth Intensive Care.

[B15] Zenz M, Piepenbrock S, Tryba M (1985). Epidural opiates: long-term experiences in cancer pain. Klin Wochenschr.

[B16] Du Pen SL, Peterson DG, Williams A, Bogosian AJ (1990). Infection during chronic epidural catheterization: diagnosis and treatment. Anesthesiology.

[B17] Crul BJ, Delhaas EM (1991). Technical complications during long-term subarachnoid or epidural administration of morphine in terminally ill cancer patients: a review of 140 cases. Reg Anesth.

[B18] de Jong PC, Kansen PJ (1994). A comparison of epidural catheters with or without subcutaneous injection ports for treatment of cancer pain. Anesth Analg.

[B19] Maier C, Kibbel K, Mercker S, Wulf H (1994). Postoperative pain therapy at general nursing stations. An analysis of eight year's experience at an anesthesiological acute pain service. Anaesthesist.

[B20] Holt HM, Andersen SS, Andersen O, Siboni K (1995). Infections following epidural catheterization. J Hosp Infect.

[B21] Ballantyne JC, Carwood CM (2005). Comparative efficacy of epidural, subarachnoid, and intracerebroventricular opioids in patients with pain due to cancer. Cochrane Database Syst Rev.

[B22] Ruppen W, Derry S, McQuay HJ, Moore RA (2006). Incidence of epidural haematoma and neurological injury in cardiovascular patients with epidural analgesia/anaesthesia: systematic review and meta-analysis. BMC Anesthesiol.

[B23] Gaul C, Neundorfer B, Winterholler M (2005). Iatrogenic (para-) spinal abscesses and meningitis following injection therapy for low back pain. Pain.

[B24] RA Moore, D Gavaghan, MR Tramèr, SL Collins, HJ McQuay (1998). Size is everything–large amounts of information are needed to overcome random effects in estimating direction and magnitude of treatment effects. Pain.

[B25] Lemeshow AR, Blum RE, Berlin JA, Stoto MA, Colditz GA (2005). Searching one or two databases was insufficient for meta-analysis of observational studies. J Clin Epidemiol.

