# Supplementary material for: Infection rates associated with epidural indwelling catheters for seven days or longer: systematic review and meta-analysis
Source: BMC Palliat Care. 2007 Apr 4;6:3. doi: 10.1186/1472-684X-6-3 (PMC1858684; doi:10.1186/1472-684X-6-3)
Supplement: Additional file 1 — Search strategy [file 1472-684X-6-3-S1.pdf]

Additional file 1: Search strategy

PubMed and Medline: all MESH terms were exploded

Intervention MESH terms: anesthesia, epidural OR analgesia, epidural OR anesthesia, spinal OR injections, spinal OR puncture, spinal OR myelography

Intervention text words: (spin* OR intraspinal OR dura* OR intradural OR epidural OR lumbar* OR theca* OR intrathecal OR subarachnoid*) NEAR (puncture* OR inject* OR anesth* OR anaesth* OR needle*)

Adverse effect MESH terms: spinal cord injuries OR epidural abscess OR hematoma

Adverse effect text words: (nerv* NEAR (injur* OR damage*)) OR ((abscess* OR infection*) AND epidural*)) OR bleed* OR hemat* OR haemat*

The search was carried out using any MESH or text words for intervention combined with (AND) any MESH or text words for adverse effect

Addition of the MESH term Epidemiologic studies
Limit: All adult: 19 years+

EMBASE: all descriptor terms were exploded

Intervention Medical Descriptor terms: anesthesia, epidural OR anesthesia, spinal OR puncture, spinal OR myelography

Intervention text words: (spin* OR intraspinal OR dura* OR intradural OR epidural OR lumbar* OR theca* OR intrathecal OR subarachnoid*) NEAR (puncture* OR inject* OR anesth* OR anaesth* OR needle*)

Adverse effect Medical Descriptor terms: spinal cord injuries OR epidural abscess OR hematoma

Adverse effect text words: (nerv* NEAR (injur* OR damage*)) OR ((abscess* OR infection*) AND epidural*)) OR bleed* OR hemat* OR haemat*
