# Supplementary material for: Infection rates associated with epidural indwelling catheters for seven days or longer: systematic review and meta-analysis
Source: BMC Palliat Care. 2007 Apr 4;6:3. doi: 10.1186/1472-684X-6-3 (PMC1858684; doi:10.1186/1472-684X-6-3)
Supplement: Additional file 2 — Details of included studies [file 1472-684X-6-3-S2.pdf]

Additional file 2: Details of included studies

Reference	Study type	Patients included	Number of patients	Technique	Duration of catheter	Objectives/remarks	Results	
							Adverse events	Comments
Aldrete 1998	Retrospective cohort	Outpatients with severe noncancer pain treated with prolonged epidural infusion	504	3164 polyamide tunneled lumbar epidural catheters with low dose bupivacaine and fentanyl	Range: 2 to 80 days<br><br>Total or median catheter-days not reported	Daily phone calls. Temperature and dressing checked twice a day. Patient to attend facility if lumbar spine pain, headache, drainage on the dressing or temperature elevation above 37.2°C experienced<br>Presence of infection confirmed by computer tomography, epidurogram or sonogram	Deep infection: 4<br>a) 2 epidural abscess<br>b) 2 fasciitis<br><br>Superficial infection: 5<br>a) 5 cellulitis	All infections treated with cefuroxime sodium every 8 hours. No patient required surgical intervention<br><br>1462 catheters inserted without antibiotic prophylaxis, resulting in 7 infections (0.4%)<br>1702 catheters inserted with prophylactic antibiotics, resulting in 2 infections (0.11%)<br><br>Staphylococcus epidermidis positive cultures in every case of infection<br><br>Onset of temperature elevation in cases of superficial infection at 11, 21, 24, 27, 29, 42 and 54 days after catheter insertion; and in case of epidural abscess at 38 and 14 days.<br>Persistent fever, headache and leukocytosis most frequently suggested infection present
Cherry 1985	Prospective cohort	Cancer pain patients	50	Percutaneous implanted port for percutaneus injections 2 to 3 times a day	Mean duration of the percutaneous implanted port: 12 weeks<br><br>Maximal: up to 9 months.		Deep infection: 0<br>(no meningitis)<br><br>Superficial infection: 2<br>(diabetic patients)	
Crawford 1983	Retrospective cohort multicentre registration study with eight major Danish anaesthesiology departments	Cancer pain patients with chronic malignant pain<br>and patients with benign chronic pain	cancer pain: 94<br>painful benign disease:11<br><br>Total: 105	No information	Mean: 65 days<br><br>Range: 7 to 283 days		Deep infection: 0<br>One patient with septicaemia with a non-fatal outcome, probably originating from some other focus. Apart from this no serious side effects. Consequently no deep infection.	Bacteriological examination of 15 catheter tips revealed 5 cultures with growth of Staphylococcus albus (without clinical signs of infection) and 1 culture with Stahyococcus aureus and concomitant septicaemia
Crul 1991	Retrospective cohort	Terminally ill cancer pain patients	110	15 patients with portable mini infusion pumps<br>95 patients received intermittent bolus injections<br><br>20 patients tunneled, 90 patients not tunneled<br><br>30 patients included with subarachnoidal morphine injections	Range: 10 to 366 days<br><br>Total treatment days: 8650 (6175 spent at home)		Deep infection: 0<br><br>Superficial infection: 5<br>a) 1 in the tunneled catheter (n = 15)<br>b) 4 in the nontunneled cather (n=95)	Oucome of 5 local skin infections not reported.<br><br>During initial period of treatment complication rate in epidural group lower than in subarachnoid group, but after three weeks complication rate in epidural group increseased dramatically (epidural fibrosis?)

de Jong 1994	Retrospective cohort	Cancer pain patients	percutaneous catheter: 98<br>ports: 52	Epidural catheters inserted under aseptic conditions in operating room.<br><br>Most percutaneous catheters not tunnelled<br><br>Antibiotic use for injection port implantation initially only for those with prior infection, then for all in last 18 months<br><br>catheters:<br>48% lumbar<br>26% lower thoracic level<br>2% upper thoracic level	Mean duration of treatment in injection port group: 23 days<br><br>Mean duration of treatment in injection group: 47 days (more than twice as long as in the port group)<br><br>Total treatment days in the nonport group: 4520<br><br>Total treatment days in the port group: 2449	Prior definitions of:<br>a) subcutaneous infection (purulent exudate from entry site and sign of inflammation of surrounding skin)<br>b) injection port infection (infection at port or somewhere along trajectory between port and epidural space)<br>c) major infection (meningitis, epidural abscess)	Deep infections: 3<br>a) 1 meningitis<br>b) 1 abscess<br>c) 1 infiltration without clear abscess<br><br>Superficial infection: no data<br><br>Overall 13.6% of catheters became infected for subcutaneous ports and percutaneous catheters<br>2.9 infections per 1000 catheter days in subcutaneous ports versus 5.9 in percutaneous catheters	Injection ports did not become infected during the first 70 days<br><br>1 patient in the percutaneous group developed clinical symtpoms of meningitis, which warranted catheter removal<br>1 patient in the terminal phase of cancer developed an epidural abscess 70 days after injection port implantation. One week later the patient became septic and died<br>1 patient with suspicion of epidural abscess, but confrmed as "infiltration" by laminectomy<br><br>Infection rate in tunnelled percutaneous catheters was 20% compared with 12% in untunelled (not significant, no numbers given)
Du Pen 1990	Retrospective cohort	Terminal disease: mostly cancer pain patients, with a few AIDS patients	cancer pain: 339<br>AIDS: 11<br><br>Total: 350	Du Pen-catheters tunnelled from superficial site	Total treatment days: 32354<br><br>Range: 4 to 1460<br><br>99,239 injections	Suspicion of deep infection investigated with pre-set definitions, investigations and procedures:<br>a) physical and neuological examination<br>b) exit site culture<br>c) aspiration of catheter and culture after removal<br>d) x-ray of epidural site with contrast medium<br>e) MRI in case of abcess<br>f) blood culture if infection systemic	Deep infection: 23<br>a) 15 epidural space infections<br>b) 8 deep catheter track infections<br><br>Superficial infection: 30<br>(exit site or superficial epidural catheter track infections)	Onset of infections varied from day 7 to day 457. Time of onset seemed not to be related to duration of catheter placement<br><br>No patients required surgical decompression, died from complications related to the epidural infections, or showed MRI or epidurogramm abnormalities after antibiotic therapy.
Erdine 1991	Prospective cohort	Cancer pain patients	225	175 patients: percutaneously implanted tunneled catheters<br><br>50 patients: subcutaneous reservoirs<br><br>156 patients: lumbar catheters<br>69 patients: low thoracic level catheters	Mean: 47.3 days<br><br>Range: 7 to 420 days		Deep infection: 0<br><br>Superficial infection: 9<br>(skin surrounding the entrance port of the catheter infected (4%))<br><br>Epidural haematoma: 2 (0.88%)	No precise comment on outcome of infected patients

Holt 1995	Prospective cohort<br>Laboratory examination of epidural catheter tips (EC)	Cancer pain patients and perioperative pain patients.<br><br>Patients with culture positive epidural catheters	Perioperative, cancer and non-cancer pain: approximately 1000	78 catheters with positive culture were examined.<br><br>Only 5 of the 78 catheters with positive cultures were tunneled.<br><br>44 catheters connected to an external injection port, 33 to an external infusion pump	Total treatement days: 11901<br><br>Duration of catheterization:<br>a) generalized symptoms: median 15 days, range 5 to 270 days<br>b) local symptoms: median 8 days, range 1 to 132 days<br>c) no symptoms: median 12 days, range 2 to 35 days		Deep infection: 11<br>(11 with central nervous system involvment, 2 of whom had epidural abscess)<br><br>Superficial infection: 36<br><br>59 of 78 patients had clinical symptoms, remaining 19 had no clinical symptoms.<br>23 of 59 patients had generalized symptoms of infection (11 had deep infection, 12 had various symptoms).<br>53 of 59 patients with clinical symptoms had superficial infections including 17 patients with deep infection generalized symptoms. So 36 (53-17) patients had a "pure" superficial infection.	147 EC tips from 1000 patients investigated, giving 78 cases with positive cultures.<br>37 of these 78 catheters used in anaesthesia and postoperative analgesia: chronic pain 41 , malignant pain 14, nonmalignant pain 27 patients.<br><br>Patients with generalized symptoms of infection had been catheterized for a longer time and were older than patients with only local symptoms.<br>48 of 59 patients (81%) developed symptoms of infection beween day 4 and day 35 after catheter insertion<br><br>No comment on outcome of patients
Maier 1994	Retrospective cohort	Perioperative pain patients	1621	Lumbar catheters : 72.6%<br><br>Thoracic catheters: 27.4%	Total treatment days: 12540<br><br>Mean: 7 days<br><br>Range: 1 to 53 days		Deep infection: 0<br><br>Superficial infection: 46<br>(one subcutaneous abscess)	No comment on oucomes<br><br>Duration >3 days did not increase risk of infection<br>Authors argue that a median duration of 4 to 7 days is necessary to achieve adaequate pain therapy. 49% of the patients needed more than 6 days
Plummer 1991	Retrospective cohort	Cancer pain patients	284 patients (313 in total, but 17 cancer patients had intrathecal therapy and 12 non-cancer patients had intrathecal morphine)<br><br>Epidurals with a port-a-cath system	Epidural catheters with subcutaneous implanted port-a-cath system	Mean duration of the port-a-cath catheter: 96 days<br><br>Range: 1 to 1215 days	Indications:<br>199: inadequate pain control<br>133: excessive side effects with conventional therapy	Deep infection: 1<br>(1 case of meningitis which occurred after the epidural catheter had been resited intrathecally)<br><br>Superficial infection: 22<br><br>All infections without sequelae following removal and/or administration of antibiotics<br>7 cases required repositioning of the epidural catheter because of infection. 19 cases required removal of port-a-caths because of infection.	

Smitt 1998	Retrospective cohort	Cancer pain patients	91 patients: 137 epidurals	All catheters inserted in the operating room under aseptic conditions.<br><br>19 patients received implanted subcutaneous port<br><br>Prophylactic antibiotics used only for subcutaneous port implantation	Total treatment days: 4326<br><br>Median patient survival: 38 days<br><br>Range survival: 1 to over 1000 days	Prior definitions of:<br>a) Superficial infection (purulent drainage or cutaneous inflammation at exit site)<br>b) Tunnel infection (inflammation along the catheter tunnel)<br>c) Pocket infection (purulent collection in subcutaneous port pocket)<br>d) Epidural space infection (epidural collection on MRI combined with isolation of microorganisms from epidural fluid or catheter tip)<br>e) Abscesses were determined by imaging	Deep infection: 12<br>(11 of whom had a spinal epidural abscess)<br><br>Superficial infection: 39	43% of patients had mild superficial infection. No tunnel or subcutaneous pocket infections. Life threating deep infections occurred in 13% of patients.<br><br>Serious deep infection present in 12 patients. 11 had epidural infection, one with meningitis. Remaining patient had abscess in paravertebral muscles of lumbar spine. The abscesses occurred after a median of 37 days (range 10 to 165). All 12 deep infections treated with intravenous antibiotics and 4 patients underwent additional decompressive laminectomy. Outcome: 3 died within 1 week after conservative therapy, remaining 9 recovered completely from their neurologic symptoms.<br>Risk factor analysis shows significant association between risk of deep infection and pump speed.<br><br>Adequate pain relief achieved in 76% for nociceptive pain and 73% for neuropathic pain.
Zenz 1985	Prospective cohort	Cancer pain patients	139	Catheter fixed 1 cm distally to the puncture site by a skin suture<br><br>139 patients had 238 catheters inserted<br><br>Lumbar: 196<br>Thoracic: 39<br>Caudal: 2<br>Cervical: 1	Total of 9716 treatment days<br>Mean: 72 days (referring to patients)<br><br>Range 1 to 700 days<br><br>34 patients treated for more than 100days, 21 for more than 150 days<br><br>26 catheters in place for more than 100 days, one for 501 days<br>Range of catheter duration 1 to 501 days		Deep infection: 2<br>(meningitis, both free of symptoms after catheter removal and antibiotic therapy)<br><br>Superficial infection: no data	
